# Healthcare System in the Kingdom of Saudi Arabia: An Expat Doctor's Perspective

**DOI:** 10.7759/cureus.38806

**Published:** 2023-05-09

**Authors:** Swathi Gurajala

**Affiliations:** 1 Medical Microbiology, Department of Respiratory Care, College of Applied Medical Sciences, Jubail, Imam Abdul Rahman Bin Faisal University, Dammam, SAU

**Keywords:** sustainable healthcare system, challenges of health care in saudi arabia, accomplishments in health care in saudi arabia, health care system in saudi arabia, global health care systems

## Abstract

Saudi Arabia has made significant progress in its healthcare system through increased healthcare spending, improved healthcare infrastructure, and better quality of care. The government has introduced initiatives such as universal health coverage, accreditation programs, and healthcare technology adoption. As a result, there has been an increase in access to healthcare services and improvements in healthcare indicators. However, the system still faces challenges such as a shortage of healthcare workers, a lack of preventive care, and health disparities between urban and rural areas. Addressing these challenges is crucial for achieving a more equitable and sustainable healthcare system in Saudi Arabia.

## Editorial

Saudi Arabia is one of the largest countries in the Middle East, with a population of more than 34 million people. The healthcare system in Saudi Arabia has undergone significant improvements over the years, with the government's significant investments in healthcare infrastructure, resulting in increased access to healthcare services across the country.

Saudi Arabia has a well-established healthcare system that provides all citizens and residents with free healthcare services. The government is responsible for providing healthcare services and is the primary funder of the healthcare system [1]. The Ministry of Health (MOH) is responsible for regulating the healthcare system and providing healthcare services across the country.

The healthcare system in Saudi Arabia consists of primary, secondary, and tertiary healthcare services. Primary healthcare services are provided through primary healthcare centers (PHCs), which provide basic healthcare services, including preventive care, health education, and screening services. Secondary healthcare services are provided through hospitals and specialty centers, which provide more advanced healthcare services, including diagnostic services, surgical procedures, and emergency care. Tertiary healthcare services are provided through specialized hospitals, which provide specialized healthcare services, including transplant services and cancer treatment.

However, despite the progress made, the system still faces numerous challenges that need to be addressed. This paper is a brief summary of the accomplishments and challenges of the Kingdom’s healthcare system.

Accomplishments of the healthcare system in Saudi Arabia

The healthcare system in Saudi Arabia has undergone significant changes and improvements over the years. The following are some of the accomplishments of the system:

Increased Healthcare Spending

The Saudi Arabian government has increased its healthcare spending significantly over the past few years. In 2020, the government allocated SAR 147 billion ($39.2 billion) for the healthcare sector, which was an increase from the previous year's budget allocation [2].

Improved Healthcare Infrastructure

The government has invested heavily in healthcare infrastructure, resulting in an increase in the number of hospitals and primary healthcare centers [3]. The government has invested in the development of healthcare facilities across the country. As of 2021, there were over 460 hospitals and 2,000 primary healthcare centers across the country [4].

Improved Quality of Care

The government has introduced several programs and initiatives to improve the quality of care, including the National Accreditation Program for Healthcare Organizations (NAHCO) and the Saudi Central Board for Accreditation of Healthcare Institutions (CBAHI) [3]. The Ministry of Health has implemented several initiatives aimed at improving the quality of healthcare services such as the National Accreditation Program for Health Facilities (NAP). The NAP assesses and accredits healthcare facilities based on several criteria, including patient safety, infection control, and quality of care. As of 2021, more than 50% of healthcare facilities in the country have been accredited [4]. The Ministry of Health (MOH) has also implemented various initiatives, such as the National Transformation Program (NTP) and Vision 2030, aimed at improving the quality and efficiency of healthcare services in the country [1].

Improved Healthcare Quality Indicators

The country has seen significant improvements in various healthcare indicators. For instance, according to the World Health Organization (WHO), life expectancy at birth in Saudi Arabia has increased from 68 years in 1990 to 74 years in 2019. Additionally, infant mortality rates have decreased significantly, from 32.5 per 1000 live births in 1990 to 5.3 per 1000 live births in 2019 [5].

Better Access to Healthcare

The government has improved access to healthcare services for its citizens. As of 2021, there were over 75,000 hospital beds, which translates to 2.3 beds per 1,000 people [4].

Introduction of Universal Health Coverage

The Saudi Arabian government introduced universal health coverage in 2019, which provides free healthcare services to all Saudi Arabian citizens [3].

*Reduction of the Prevalence of Communicable Diseases* 

The government has implemented several programs to control and prevent communicable diseases, including the National Immunization Program (NIP) and the Saudi Antimicrobial Resistance Surveillance System (SARSS) [1].

Adoption of Healthcare Technology

The government has invested in healthcare information systems, electronic health records, and telemedicine. This has improved the efficiency of healthcare delivery and made it easier for patients to access healthcare services.

Challenges facing the healthcare system in Saudi Arabia

Despite the progress made, the healthcare system in Saudi Arabia still faces numerous challenges. The following are some of the challenges facing the system.

Shortage of Healthcare Workers

There is a shortage of healthcare workers in Saudi Arabia, particularly in rural areas. The majority of healthcare professionals are concentrated in urban areas, resulting in underserved rural areas [1]. According to a report by the Saudi Arabian General Investment Authority (SAGIA), the country has a deficit of around 15,000 doctors and 20,000 nurses [2]. This shortage has resulted in longer wait times and reduced access to healthcare services [3]. The government has implemented several initiatives to address this challenge such as the establishment of new medical schools and the recruitment of foreign healthcare professionals.

Lack of Preventive Care

The healthcare system in Saudi Arabia focuses more on curative care than preventive care. This has resulted in higher rates of non-communicable diseases (NCDs) such as diabetes, cardiovascular disease, and obesity. NCDs are the leading cause of mortality in the country, and their prevalence is increasing. The government has implemented several programs to address this issue, including the National Program for Prevention and Control of Diabetes and the Saudi Heart Association [3]. However, more needs to be done to reduce the prevalence of NCDs and improve the health outcomes of the population.

Limited Private Health Coverage

MOH statistics indicate that employers are typically the primary providers of private health insurance policies in Saudi Arabia. Employees and their dependents are often covered under these schemes as part of their employment benefits. The schemes accounted only for around 67% of all private health insurance policies in 2020. Hence, more than 30% of the population relied on public healthcare services provided by the MOH. This has resulted in overcrowding in public healthcare facilities and longer wait times [4].

 *Limited Mental Health Services*

Mental health services in Saudi Arabia are limited, and there is a stigma associated with mental health issues. Because of the cultural attitudes toward mental health as well as a lack of awareness and understanding about mental illness, mental health issues have been underreported significantly in the country. Also, another challenge is the shortage of mental health professionals, particularly in rural areas. According to the WHO, there are only 2.2 psychiatrists per 100,000 people in Saudi Arabia, which is significantly lower than the global average of 9.0 psychiatrists per 100,000 people. This has resulted in a lack of access to mental healthcare services [5].

Restricted Healthcare Financing

The healthcare system is primarily funded by the government, and there is a need to explore alternative financing mechanisms to ensure the sustainability of the healthcare system [1].

In conclusion, despite significant progress, Saudi Arabia's healthcare system still has a long way to go to provide quality healthcare to all citizens. Addressing the challenges facing the healthcare system will require a coordinated effort from all stakeholders. The government, healthcare professionals, and the private sector need to work together to ensure that the healthcare system in Saudi Arabia can meet the growing demand for healthcare services and provide quality care to all citizens. To provide quality healthcare services across the country, the government needs to invest in training and educating healthcare professionals. Additionally, to address the high prevalence of non-communicable diseases and mental diseases, the government needs to implement more effective preventive programs and initiatives. Furthermore, to ensure the sustainability of the healthcare system, the government needs to explore alternative financing mechanisms. This can include implementing health insurance schemes or public-private partnerships to increase funding for the healthcare system. The private sector can also play a significant role in addressing the challenges facing the healthcare system by investing in healthcare infrastructure and providing healthcare services.
